# Living With Persistent Respiratory Symptoms of Long COVID: Qualitative Study Among Brazilian Adults 12 Months After Acute Infection

**DOI:** 10.1111/hex.70409

**Published:** 2025-09-23

**Authors:** Giovana Alves Santos, Carlos Laranjeira, Lígia Carreira, Vanessa Denardi Antoniassi Baldissera, Maria Fernanda do Prado Tostes, Viviani Camboin Meireles, Rosella Santoro Ageno, Maria Aparecida Salci

**Affiliations:** ^1^ Department of Postgraduate Nursing State University of Maringá Maringá Brazil; ^2^ School of Health Sciences, Campus 2 Polytechnic University of Leiria Leiria Portugal; ^3^ Centre for Innovative Care and Health Technology (ciTechCare) Polytechnic University of Leiria Leiria Portugal; ^4^ Comprehensive Health Research Centre (CHRC) University of Évora Évora Portugal; ^5^ Universidad de Antofagasta Antofagasta Chile

**Keywords:** adult, grounded theory, nursing care, post‐acute COVID‐19 syndrome

## Abstract

**Introduction:**

The majority of those infected with COVID‐19 undergo a brief duration of clinical illness. In certain instances, symptoms endure for months or years after the initial viral exposure—a condition characterized as Long COVID (LC). The experience of this illness remains largely unexplored as it has only recently surfaced. This study aims to understand the repercussions of persistent respiratory post‐COVID symptoms in Brazilian adults 12 months after SARS‐CoV‐2 infection.

**Methods:**

A constructivist grounded theory study was employed. Data were collected through individual interviews with adults with persistent respiratory symptoms of Long COVID in Brazil. Data collection took place between September 2023 and February 2024. Data analysis was performed on a constant comparative basis and concurrent with data collection to understand the findings.

**Results:**

Twenty‐four individuals (12 females, 12 males) with a median age of 43.29 ± 9.09 years participated. The data analysis generated a central category—living with the long‐term effects of COVID‐19: breathlessness pervades everything—around which three categories emerged: (1) imbalance between life before and after being infected by COVID‐19; (2) living with acute post‐COVID respiratory symptoms; and (3) struggling with persistent post‐COVID respiratory symptoms.

**Conclusion:**

Our analysis of the perceived needs of individuals with Long COVID underscores the urgent necessity for legislative reform to acknowledge LC as a disability that requires clear diagnostic criteria. Approaches to treatment and rehabilitation are required to evaluate the extent to which functioning and disability improve. Lastly, this study highlights the complex problems encountered by individuals with Long COVID, including employment uncertainties, everyday tasks and social relationships.

## 1 Introduction

With the advent of globalization, the speed with which diseases spread internationally has been modified [1]. On March 11, 2020, the World Health Organization (WHO) declared the beginning of the coronavirus disease 2019 (COVID‐19) pandemic, a disease caused by SARS‐CoV‐2 [2]. The end of the pandemic was officially declared by the WHO on May 5, 2023, as the disease no longer met the criteria of an extraordinary event [3]. However, it was clear that this condition continued to deserve the necessary attention regarding the care given to people [3]. By the end of January 2025, the total number of accumulated cases of COVID‐19 globally was around 777 million confirmed cases and seven million deaths [4].

The COVID‐19 pandemic has impacted people's lives in biological, socioeconomic, environmental and psychological aspects, bringing changes to people's daily routines [5]. Globally, its impact was heterogeneous in different populations, mainly affecting the most vulnerable populations that presented a greater risk of developing Long COVID (LC) [6, 7, 8, 9]. Likewise, the population of disabled individuals unable to work due to LC has risen significantly, resulting in considerable economic expenses [10, 11]. LC presents significant issues for global and public health [12]. Notwithstanding this urgency, the extensive ramifications of LC have thus far been ignored and insufficiently studied.

The National Academies of Sciences, Engineering, and Medicine (NASEM) defined LC as a chronic condition associated with prior SARS‐CoV‐2 infection and lasting at least 3 months. Its onset may occur immediately after the acute COVID‐19 infection or weeks or months after acute infection [13]. Symptoms of LC usually appear 12 weeks after acute infection (aiming to elucidate if COVID‐19 may become chronic). LC may involve single or multiple symptoms, with different levels of severity, ranging from mild to severe symptoms [13]. Currently, the existing research indicates that differentiation between post‐COVID and extended COVID is ambiguous, mostly because it is based merely on a temporal assessment [14]. Fernandez‐De‐Las‐Peñas et al. [15] clarified the timeframe used for defining post‐COVID symptoms. The authors differentiate between ‘acute post‐COVID symptoms that occur between the 5th and 12th week after acute infection; long post‐COVID symptoms that appear between the 12th and 24th weeks (Long COVID); and persistent post‐COVID symptoms, which last more than 24 weeks and can be called Long Persistent COVID’ [15]. However, LC is an emerging disease characterized by significant unknowns, with numerous patients lacking evidence of infection due to different factors, such as inaccessible testing.

A recent review estimated the frequency of LC as between 50% and 85% for unvaccinated people who were hospitalized, 10% and 35% for unvaccinated people who were not hospitalized and 8% and 12% for vaccinated people [16]. The prevalence of Long Persistent COVID after 2 years is around 30%. Among the most common symptoms are respiratory symptoms, such as fatigue, shortness of breath, cough, tiredness and dyspnoea [17, 18, 19, 20, 21, 22, 23, 24].

Evidence shows that people struggling with these persistent symptoms refer to themselves as ‘long haulers’ [25]. The symptoms are heterogeneous and cyclical, with affected people seeking to self‐manage the symptoms of the disease, given the frequent undervaluation of symptoms by health professionals [26, 27]. Despite the increasing medical evidence regarding LC, patients encounter stigma, insufficient support, challenges in accessing services and obtaining diagnoses, as well as misunderstanding, dismissal and disbelief regarding their symptoms and knowledge [28, 29, 30, 31].

The literature also highlights that people with LC perceive their symptoms as complex and having an effect on daily life, determining a nonlinear health‐disease trajectory [32]. Thus, LC is a multisystemic disease that has a complex nature, often due to the lack of a formal diagnosis, marked by uncertainty and inadequate care after discharge, making the transition process more challenging [16, 33, 34, 35, 36]. People with LC may experience ambivalent feelings related to the disease and several uncertainties about the future [16, 33, 34, 35, 36]. Recent qualitative systematic reviews on LC underscore the extensive issues encountered by individuals with lived experience, encompassing persistent symptoms, mental anguish, fluctuating social support and obstacles to healthcare access [26, 27, 37].

In this sense, healthcare systems must be prepared to address LC, a syndrome defined by an extended clinical trajectory. While access to healthcare services is essential for safeguarding the well‐being of impacted people, in Brazil there are substantial obstacles to healthcare access and healthcare professionals are considerably deficient in understanding this ailment [28, 35, 38]. The inadequate availability of rehabilitation units and the absence of training programs for professionals, particularly in primary care, may lead to increased healthcare costs and further obstruct access to care, as patients are evaluated or referred solely based on their symptoms without a holistic or comprehensive approach [39].

Given that respiratory symptoms were one of the most prevalent symptoms in people with LC [19, 20, 21], and noticing a lack of studies carried out in Brazil that specifically investigate the repercussions of LC in people with respiratory symptoms, this study's aim was to understand the repercussions of persistent respiratory post‐COVID symptoms in Brazilian adults 12 months after SARS‐CoV‐2 infection. Due to the nascent nature of LC as a research subject, access to the experiences of LC patients remains scarce. We hope that this study will advance the understanding of LC experiences beyond mere biomedical facts, reducing the risk of LC becoming an ‘invisible’ or ‘silent’ pandemic [37].

## 2 Methods

### 2.1 Study Design

This study is part of a larger research project entitled ‘Longitudinal Monitoring of Adults and Elderly People Discharged from Hospital Due to COVID‐19’, developed in a cohort of people who developed COVID‐19 and resided in the state of Paraná, Brazil [40].

In this study, a qualitative approach was used, based on Charmaz's Constructivist Grounded Theory Grounded Theory (CGT) [41]. This theory consists of a method of conducting qualitative research that focuses on the creation of conceptual schemes of theories. The chosen CGT prioritizes the phenomena under study and sees both data and analyses as having been generated from shared experiences and relationships with the participants [41, 42, 43]. Charmaz [41] elucidates how CGT can be used to examine several chronic illnesses (e.g., cardiovascular diseases, diabetes, fibromyalgia, among others), with regard to their significance, impact on self and identity, and temporal aspects. The CGT approach is particularly appropriate for examining the personal and contextual aspects during the experience of a novel and unpredictable disease such as LC.

This study was conducted and reported following the COREQ (Consolidated Criteria for Reporting Qualitative Research) checklist [44].

### 2.2 Setting and Participants

Brazil has a Unified Health System (SUS) as its public health system, in which all citizens have the right to free public health care. In terms of healthcare services, both public and private choices exist, with 70% of the population depending only on SUS, especially for primary healthcare (PHC) [45]. The study was carried out in the state of Paraná, located in the southern region of Brazil. As other states, Paraná exhibited one of the highest COVID‐19 transmission rates necessitating hospital admissions [46], hence warranting the recruitment of subjects in this state who developed LC.

Potential participants were selected using a purposive sampling technique, using records of individuals who developed acute COVID‐19 and were registered in the ‘Notifica COVID Paraná’ and ‘Influenza Epidemiological Surveillance Information System (SIVEP‐Gripe)’ databases. Both are obligatory epidemiological surveillance datasets for COVID‐19: the state‐specific information system for Paraná, known as Notifica COVID‐19 Paraná, which encompasses outpatients or mild cases of the disease, and SIVEP‐Gripe which includes hospitalizations for severe acute respiratory syndrome (SARS) due to COVID‐19 (both severe and moderate cases) [47].

The following inclusion criteria were defined: (a) age ≥ 18 years and < 60 years; (b) tested positive for SARS‐CoV‐2 using reverse transcription polymerase chain reaction (RT‐PCR), between September 2022 and February 2023 and reported in the ‘Notifica COVID Paraná’ and SIVEP‐Gripe databases; (c) self‐reported persistent respiratory symptoms 12 months after the acute infection and without another justifying cause; and, (d) responded to the telephone contact (up to three attempts). Pregnant or postpartum women during the period of acute COVID‐19 infection and participants without cognitive conditions were excluded.

The participants in the study were not formally diagnosed with LC, so this was not considered an inclusion criterion. This decision is due to the fact that the diagnosis of LC is challenging, given the limited knowledge of health professionals regarding the diagnosis and the contrast between the high prevalence of reported LC symptoms and the low frequency of formal diagnoses [48].

To obtain maximum sampling variation, individuals were recruited regardless of sex and race. The total number of participants was not pre‐established; rather, it emerged from a methodical and theoretical sampling approach. The initial 15 participants were chosen using a purposive sample method, and a preliminary study of their experiences enabled the identification of essential categories or concepts [49]. Theoretical sampling was utilized to complete data collection, with concurrent analysis guiding the selection of relevant data and shaping the evolving theory [41]. Sampling was concluded upon reaching theoretical saturation, as no more insights were obtained from the data [41]. The total sample consisted of 24 participants.

### 2.3 Data Collection

Data collection took place between September 2023 and February 2024. Telephone contacts were extracted from registration platforms (Notifica COVID Paraná and SIVEP‐Gripe). The first contact with the participants was made via WhatsApp. After acceptance, telephone interviews were scheduled according to the participant's availability. The interviews were guided by a semi‐structured script, based on previous evidence [50, 51] and validated by a group of four experts in qualitative research and experienced nurses. The interview script consisted of open‐ended questions that addressed the persistence of respiratory symptoms after COVID‐19 and their repercussions on the subject's daily life. Probing questions were also used to explore and clarify themes, such as ‘Tell me more about that’; ‘Could you give me an example?’

The interview script was modified throughout the data collection as new themes emerged, meeting the assumptions of CGT [41]. Data collection was carried out by the first author (G.A.S.), a registered nurse with clinical experience and previous experience in conducting interviews. To reduce social desirability bias [52] and response bias [53], a nonconfrontational and respectful approach was adopted during the interviews, using some communication techniques such as questioning (e.g., open‐ended questions, requesting examples), reformulation, synthesis and the use of silences during the interviews to encourage interviewees to express themselves. The researcher was not involved in the care of the participants.

Field notes on the researcher's perceptions were taken during and after the interviews [41]. The interviews lasted on an average of about 30 min (range: 20–50 min) and were audio recorded and transcribed in full. There were no repeat or follow‐up interviews.

### 2.4 Data Analysis

Data collection and analysis were performed simultaneously using a constant comparative approach during this stage. First, initial coding was carried out. The data were then coded inductively, line by line, incident by incident, with emphasis on important concepts and phrases. Subsequently, focused coding was carried out to thoroughly analyse the data obtained. Finally, theoretical coding was carried out, highlighting the central category and the relationships with other categories [54]. Frequent comparison between the coded concepts generated the central category, identified through an iterative process over several meetings with the co‐researchers. Finally, integration was carried out, which developed the analytical process until all categories were saturated, supporting the phenomenon. MaxQDA software (version 2024) was used to manage and archive everything from raw qualitative data to coding. Quotes from participant narratives were presented according to interview order (e.g., P1, P2,…) and age.

### 2.5 Rigour

The research met methodological rigour following the criteria of credibility, originality, resonance and usefulness, as proposed by Charmaz [54, 55]. Credibility was ensured by the careful way in which the interviews were transcribed, with detailed notes of the researcher's perceptions during data collection, and also with the theoretical saturation of the data, when the selected participants stopped providing new information. Originality was guaranteed, with the construction of memos, diagrams and literature research contributing to the researchers’ reflexivity process in the production of original discoveries [55, 56, 57]. The research team included nurses with clinical experience in managing people living with chronic conditions (G.A.S. and R.S.A.) and researchers with expertise in qualitative health research (C.L., L.C., V.D.A.B., M.F.P.T., V.C.M. and M.A.S.). Resonance was achieved because the researchers arrived at concepts that accurately reflect the experiences of the participants and are transferable to similar contexts. Finally, the usefulness criterion covered the ability to deepen research participants’ understanding of their experiences, and whether the study could improve their daily lives and thus assist in the development of good care practices.

### 2.6 Ethical Issues

The study followed the ethical requirements proposed by the Declaration of Helsinki and was approved by the Research Ethics Committee of the State University of Maringá (UEM) (Opinion No. 4,165,272). Before each interview, informed verbal permission was obtained, including agreement to audio recordings. Participation was voluntary and participants did not receive any type of compensation. Participants were informed that they could choose to leave the study at any time without prejudice.

## 3 Results

### 3.1 Sample Description

The sample included 24 people, aged between 26 and 59 years (Table 1). Half of the sample was female. The majority were White (*n* = 16), had more than 9 years of education (*n* = 20) and were employed (*n* = 22). Ten participants received treatment in intensive care units due to severe SARS‐CoV‐2 infection; the rest were admitted to the ward for moderate infection (*n* = 14). The majority (*n* = 13) reported having some chronic illness and mentioned having only used private health services during the 12 months following acute COVID‐19 infection (*n* = 10).

**Table 1:** Description of participants (*N* = 24).

Variables	Adults with LC respiratory symptoms
Age	
Mean ± SD (range)	43.29 ± 9.09 (26–59)
Sex, *n* (%)	
Male	12 (50)
Female	12 (50)
Race, *n* (%)	
White	16 (67)
Non‐White	8 (33)
Severity of acute SARS‐CoV2, *n* (%)	
Moderate (Medical unit)	14 (58)
Severe (intensive care unit)	10 (42)
Education, *n* (%)	
≤ 9 years	4 (17)
> 9 years	20 (83)
Employment status, *n* (%)	
Employed	22 (92)
Unemployed	1 (4)
Retired or pensioner	1 (4)
Chronic disease, *n* (%)	
Yes	13 (54)
No	11 (46)
Use of health services, *n* (%)	
Public system	8 (33)
Private system	10 (42)
Public and private systems	6 (25)

### 3.2 Overview of Findings

The data analysis generated a central category—living with the long‐term effects of COVID‐19: breathlessness pervades everything—around which three categories emerged: (1) imbalance between life before and after being infected by COVID‐19; (2) living with acute post‐COVID respiratory symptoms; and (3) struggling with persistent post‐COVID respiratory symptoms. During the interviews, it was possible to observe the repercussions of respiratory symptoms and people with Long Persistent COVID. It was possible to see a timeline that evolves from the pre‐ to the post‐COVID‐19 phase. Although the pandemic as a health emergency has come to an end, COVID‐19 has left its mark and brought changes to people's lives. A summary of the findings is reported as follows in Table 2.

**Table 2:** Description of categories and subcategories.

Category	Subcategories
Imbalance between life before and after being infected by COVID‐19	Before I was happy and didn't know it!
	Feeling different: breathlessness became a problem
Living with acute post‐COVID respiratory symptoms	Noticing post‐COVID respiratory symptoms and trying to return to normalcy
	Challenges in returning to work
Struggling with persistent post‐COVID respiratory symptoms	Daily fluctuation of symptoms and feeling isolated and stigmatized
	Learning from experience and designing the future
	Challenges in accessing care

#### 3.2.1 Imbalance Between Life Before and After Being Infected By COVID‐19

This category highlights how SARS‐CoV‐2 infection marked a turning point in the lives of participants. This category is anchored in two subcategories: (1) before I was happy and didn't know it!; and (2) feeling different: breathlessness became a problem.

##### 3.2.1.1 Before I Was Happy and Didn't Know It!

Participants reported that before COVID‐19 they had a more active life. However, with the infection, respiratory symptoms appeared that had an impact on their lives. It is worth noting that life gained new meanings that made them rethink life before the infection.

My life before was much more peaceful, I was a person without fear, without traumas. I was happy and didn't know it.

(P19, 43 years old)

I cycled to work, just as I liked. I went everywhere by bike, to the bank and to the market. After the infection, I almost stopped leaving the house, because I couldn't.

(P7, 45)

Look, my life before was more active, I had more availability to do things. I used to go out more, I used to go to my parents’ house, now I just stay at home… how happy I was.

(P24, 40 years old)

I was living very well before COVID, I didn't have the symptoms I had afterwards.

(P23, 32 years old)

##### 3.2.1.2 Feeling Different: Breathlessness as a Problem

Most of the participants highlighted how the infection has taken away their ability to carry out their daily activities effortlessly. Dyspnoea affected other abilities, notably the ability to react and memorize.

I'm in college and before I would read a book, something, I managed to remember a lot of things, now I read, I have to read it three times to be able to get something out of it, to reason things out takes longer, it seems like it didn't before, before I would read, quickly, do some work and it was much calmer.

(P9, 44 years old)

Before I was electric to do things, I did them, I finished them quickly, (…). As I said, before I was more electric, now I'm slower, but otherwise I'm normal.

(P18, 57 years old)

Although respiratory symptoms were a source of concern, they were not considered by the doctor as secondary to COVID‐19. I was in the process of studying for a master's degree and at the time I was about to leave isolation, I started to feel worse, especially when breathing. It was COVID that comes and knocks you down, there was a doctor who just said it was anxiety, because the symptoms were similar.

(P22, 29 years old)

In one case, a participant mentioned how the iatrogenic effect of mechanical ventilation in the acute phase of COVID‐19 caused significant respiratory complications.

*They discovered that I had tracheal stenosis, as I was intubated, the intubation injured my trachea. So, I had 98% of my trachea compromised, which is why I had a lot of difficulty breathing* (P1, 26 years old).

#### 3.2.2 Living With Acute Post‐COVID Respiratory Symptoms

Following acute COVID‐19 symptoms, participants reported persistent respiratory symptoms during the first 3 months after acute infection. This category was based on two subcategories: (1) noticing post‐COVID respiratory symptoms and trying to return to normality and (2) challenges in returning to work.

##### 3.2.2.1 Noticing Post‐COVID Respiratory Symptoms and Trying to Return to Normality

Participants report persistent respiratory symptoms after the acute phase, in the form of dyspnoea and fatigue. Symptoms varied in intensity, from fleeting and easily controllable to more severe occurrences. These intrusive symptoms generated dependence and a consequent need for support in carrying out daily life activities.

It was all with someone's help, to take a shower, and it's still like that today, since then I've continued to need help with that. (P8, 41 years old) Everyday things, like hanging clothes, any slightly greater effort, would cause me shortness of breath (participant coughing).

(P12, 51 years old)

I remember that the shortness of breath never went away. There were times when the shortness of breath was minimal, but others when it was very intense. That's why my daughter came and helped me a lot.

(P24, 40 years old)

I'm a singing teacher, I depend on my voice. So, the impression I had was that I was always tired. I was permanently tired of doing anything, of singing, of talking. Sometimes even to climb the stairs of my building.

(P20, 45 years old)

In an attempt to recover pre‐COVID‐19 normality, some participants sought adjustments to reduce the impact of respiratory symptoms on their daily lives, highlighting the importance of family and health professionals in this process.

Little by little I tried to be able to do things, otherwise I would be dependent on my family, and I didn't want that.

(P3, 50 years old)

It was a difficult period, but my family and professionals were in favor of my recovery, even though I couldn't do anything.

(P2, 42 years old)

The days went by, but the tiredness didn't diminish and I ended up having to use the fan at home, during the night, to be able to do something. Even with the respiratory issue, I didn't stop doing anything. I believe that because I have such a stubborn personality, even with difficulty, even if I took more breaks, it took a little longer to do these family and domestic activities, but I did them.

(P22, 29 years old)

##### 3.2.2.2 Challenges in Returning to Work

There were several challenges in returning to work, whether due to reports of absenteeism due to respiratory symptoms, feelings of guilt for not being able to accomplish previous activities, decreased productivity or facing potential financial strain.

During the first three months, I feel… I feel a bit guilty; I can't be as productive. I was afraid of having financial difficulties because I couldn't work in the same way.

(P17, 59 years old)

When I started working, I had to see fewer people, until the breathing exercises (rehabilitation) that I was doing started to show results, but I became much less productive during that period.

(P15, 33 years old)

Due to the fatigue I had, I worked the bare minimum. I was the coordinator of a shelter institution, I reported directly to the judiciary. So, my focus was on that, which was to respond to the judiciary, and other demands were left behind. It was the bare minimum.

(P22, 29 years old)

However, in a few cases, work contexts were sensitive to changing the worker's role to adapt to the new health condition.

I was hospitalized for ten days, after that I had a month's sick leave and then vacation, because I was very limited and couldn't work. When I started working, I got very tired, to the point that I had to change jobs so I wouldn't get so tired.

(P13, 43 years old)

#### 3.2.3 Struggling With Persistent Post‐COVID Respiratory Symptoms

After the first 3 months postinfection, participants reported that they still perceived the influence of persistent respiratory symptoms. This category was anchored in three subcategories: (1) daily fluctuation of symptoms and feeling isolated and stigmatized; (2) learning from experience and designing the future; and (3) challenges in accessing care.

##### 3.2.3.1 Daily Fluctuation of Symptoms and Feeling Isolated and Stigmatized

Many participants reported an undulating trajectory of symptoms in which at certain times the symptoms appeared to improve and at others they intensified, significantly interfering with their lives.

There are periods when I feel better, but lately I feel that the shortness of breath is returning to what it was before.

(P15, 33 years old)

In my day‐to‐day life, I even walk relatively well. But when I walk a lot or do any other activity, I feel like I get very short of breath.

(P12, 51 years old)

It's quite aggravating, because I cycle, I swim, so I feel it a lot during my activities. I notice that the evolution is very inconsistent.

(P14, 47 years old)

Some participants reported that they noticed a decrease in their immunity and with that a greater vulnerability to becoming ill or manifesting exacerbated symptoms.

Since I was infected with COVID‐19, I get sick more easily, I develop flu‐like symptoms and I have difficulty breathing.

(P19, 43 years old)

I noticed that I am much more sensitive now, any little problem causes a cold and flu even after taking the vaccine, I don't know how this can be.

(P13, 43 years old)

In fact, I was left with after‐effects from COVID‐19, it's complicated because I developed diabetes, something I didn't have.

(P7, 45 years old)

In addition to the physical repercussions, participants also experienced the impacts of symptoms on the social domain of their lives, through reduced social interactions and family activities.

Today it is more limited, any family activity that I have and that requires a bit of breathing I have more difficulty with.

(P8, 41 years old)

I think that maybe because of the tiredness and shortness of breath that I still feel, I am more antisocial.

(P21, 31 years old)

I feel much more tired, which doesn't allow me to have the life I used to have. I use an oxygen mask and it makes me lose control over my life.

(P19, 43 years old)

I try not to make so much of an effort, to do things more slowly… Before I wanted to do everything quickly. But today it is no longer like that. I was no longer able to participate in social activities that I enjoyed, such as going for walks and running. Now I rarely leave the house, because I can't.

(P4, 56 years old)

Two participants felt that they were judged as being responsible for their condition. As a result, they feel stigmatized because when seeking help, they feel unseen because sometimes professionals do not value their symptoms. At the same time, the public health system is limited in the responses it offers, justifying the search for alternative solutions.

I sought help at the public health unit, but in addition to the wait, I feel that they don't always appreciate my shortness of breath. I saw a news report where they talked about long COVID, they never confirmed this diagnosis for me. Then I changed municipalities and had to start a new process, but the health center of the municipality where I came to live is even more precarious, and I didn't go there anymore.

(P23, 32 years old)

When I go to the appointment, the doctors ask if I had COVID, and they end up not valuing my complaints, by not going deeper into some complaints. I had to go private to get answers.

(P5, 33 years old)

##### 3.2.3.2 Learning From Experience and Designing the Future

In many cases, participants sought to reframe everything that had happened in their lives in the 12 months postinfection. The respiratory symptoms of persistent LC represented a turning point, because they did not return to their pre‐pandemic health situation, they changed their lifestyles and began to see life differently.

I'm managing to exercise more, I'm managing to take care of myself a little more than before the pandemic and during the pandemic.

(P15, 33 years old)

In a way I became a little healthier because I started to control things more. It's different, you have another way of looking at things.

(P17, 59 years old)

I can say that in my case, COVID was a serious case, I was intubated for a long time, and in addition to the permanent shortness of breath, I forgot how to walk, talk, eat, I had a tracheostomy, so I became very weak, that's why I say that I was born again. In my mind, this is a second chance. So, I'm taking more time to enjoy time with my family. To stroll. Enjoy leisure time with your family. I was a much more explosive, more energetic guy. Today I am a much more withdrawn, calmer guy.

(P2, 42 years old)

Notably, participants with no pre‐existent chronic illness expressed wanting to practice better health behaviours, as illustrated by P23: *I think that to avoid worsening I have to do more physical exercise and eat better to be able to deal with the symptoms. Maybe then things can get better*.

Two participants with previous chronic conditions revealed that they were more concerned about managing other health challenges, such as high blood pressure or hyperglycaemia.

With long COVID I have to be more concerned about my blood pressure. I heard about a neighbor who had COVID‐19 and had a stroke due to hypertension.

(P3, 50 years old)

My diabetes has never been under control, but now with this COVID thing, I'm afraid it will get worse. I have to be more careful.

(P18, 57 years old)

##### 3.2.3.3 Challenges in Accessing Care

Some participants anticipated that healthcare providers need to invest time in comprehending their conditions and actively engage in their treatment process. Sometimes, people experience frustration due to a lack of knowledge and empathic communication regarding LC.

When I go to the doctor and tell him that I had COVID and that I now have difficulty breathing, I realize that he doesn't value what I say.

(P2, 42 years old)

Knowledge about long COVID is still limited. I see that professionals have many questions about this condition.

(P3, 50 years old)

Moreover, one participant often encountered difficulties in timely access to therapies and occasionally resorted to the emergency department, particularly for lengthy COVID symptoms that healthcare practitioners did not regard as severe (e.g., shortness of breath, exhaustion).

I continue to have shortness of breath and go to the emergency room to deal with the symptoms. I also have already spoken to the doctor about doing physical therapy, but the waiting time is very long… and the disease continues.

(P4, 56 years old)

## 4 Discussion

To our knowledge, this is the first qualitative study conducted in Brazil on the experiences of people with persistent respiratory post‐COVID symptoms and how they perceived the impact of these symptoms on their lives. The findings point to an imbalance between life before and after being infected by COVID‐19, where breathlessness became a problem. In parallel, the findings portray what it is like to live with acute post‐COVID respiratory symptoms and the challenges in returning to work. Finally, participants suffering from persistent LC recognize themselves as vulnerable, feeling isolated and stigmatized, trying to learn from their experience and plan for the future.

In line with this study's findings, evidence suggests that respiratory symptoms stand out among the symptoms of LC with the greatest impact on the functioning of affected people, given the impact on daily activities, as well as the physical, emotional and social consequences [58, 59]. Additionally, findings from a naturalistic qualitative investigation reveal that certain patients suffering from protracted COVID encounter challenges in resuming pre‐infection habits and activities due to the ongoing physical and mental health symptoms that hinder everyday functioning [60]. Some studies have shown that for some people, symptoms of LC lessen over time, while for others, symptoms fluctuate over time [61].

In the current study, participants reported some difficulties associated with the presence of respiratory symptoms of LC, highlighting the importance of family and support networks in their recovery process. Likewise, persistent symptoms impacted professional life, generating a decrease in productivity and difficulties in concentration, justifying periods of rest between activities [62]. For some, lack of efficiency at work led to feelings of guilt and absenteeism [63, 64]. Another concern was related to the decrease in productivity and the challenges imposed by the impact of LC in the work context, with the potential to generate financial limitations [65]. Previous studies have already reported the need to manage persistent symptoms by establishing deadlines for returning to work, seeking answers and accessing health services [28, 66]. The findings highlight that participants experienced uncertainty, fear and identity threat, particularly the fear that recovery might not be possible [25]. Similar to McEwan et al. [67], our findings underscore the necessity of addressing substantial deficiencies in support networks for people with LC by linking them to resources for financial aid, mental health services and job accommodation requests.

Additionally, there have been reports of ambivalence in the presence of persistent post‐COVID symptoms. As reported by Wurz et al. [34], participants mentioned marked changes in their functional capacity and ability to maintain their responsibilities, given that they were no longer able to care for their homes, families, and in some cases themselves, due to persistent respiratory symptoms. The literature shows that people with LC have a lower quality of life when compared to people who have not had the disease [68]. Possible explanations include lung impairment, fatigue, muscle pain and anxiety [69]. In our study, people with LC recognized themselves as more vulnerable both physically and psychologically. Evidence suggests that individuals with Long Persistent COVID have a dysregulated immune response [70, 71], which explains the presence of persistent symptoms and the reactivation of pre‐existing conditions [70].

In parallel, accessing health services can be long and challenging [62]. The absence of service availability and restricted treatment alternatives fosters a sense of pessimism regarding recovery, adversely affecting mental health and overall well‐being [25, 29, 72]. Qualitative investigations have elucidated the arduous experiences encountered by individuals with LC in obtaining healthcare services, encompassing challenges in securing a diagnosis, manoeuvreing through services, and being regarded seriously by healthcare providers [26, 34, 73]. Lack of knowledge about the disease can generate anxiety and confusion, and this is accentuated when health professionals offer divergent and conflicting guidance [26]. Our findings align with prior qualitative studies indicating a widespread lack of comprehension of LC [26, 29]. This leads to experiences of judgement and stigmatization in society, as well as a deficit in belief or empathy from employers and general practitioners, which exacerbates the feelings of burden and loneliness linked to LC [25, 74]. It has also been highlighted that treatment is often significantly targeted at the signs and symptoms of the disease, rather than following a comprehensive, person‐centred care model [75].

Even with all the difficulties experienced, participants with persistent post‐COVID symptoms sought to find meaning in their experiences and acquire new perspectives for the future. The literature shows that after COVID‐19, people face uncertainty about recovery and must deal with persistent post‐COVID symptoms [76]. Furthermore, faced with uncertainties about the future, they need to adjust to a new normality in the face of physical, economic, emotional and social challenges that were already present in their lives [77].

### 4.1 Strengths and Limitations

CGT seeks to uncover a co‐constructed world rather than an objective truth [41]. Consequently, CGT brings a contextual comprehension of phenomenon under study [41, 78]. This study's significance resides in unveiling categories and patterns within a specific case/context that enhances theoretical understanding of meaning in life amidst chronic illness; in its transferability, allowing rich descriptions; and in providing interpretations that prompt readers to contemplate contextualized findings pertinent to their own situations [78]. Transferability was contextualized to elucidate how individuals with a novel illness perceive meaning in life during a unique pandemic and to provide narrative tools for a clearer understanding of patients’ life‐changing experiences.

The transferability of our data is limited to a single area of Brazil and may not reflect the reality of other Brazilian states, given that Brazil has a very large geographical area with different social and health resources available. Telephone interviews were considered the most appropriate approach to ensure access and participation in the study. This interview method, although biased due to the absence of nonverbal cues and decreased spontaneity, was chosen to reliably capture participants’ experiences. As this study used self‐reporting as a data source, memory bias was present, but we sought to mitigate its emergence by formulating additional questions and communication techniques (e.g., reformulation, synthesis, summary, questioning) that would support the interview. Also, because our research uses self‐reporting, it does not have other sources of information, such as exams or the participant's health history, which could clinically confirm the diagnosis of LC. LC symptoms can vary widely between individuals and even within the same individual over time. This makes it challenging to develop a standardized approach for tracking symptom severity and progression. Ultimately, this study did not examine the influence of participants’ medical histories on the outcome, including COVID‐19 vaccination status, which may be seen as a potential limitation. Subsequent research should investigate this domain and the extent to which these factors affect other health outcomes.

### 4.2 Implications for Practice

Research on LC is limited, so identifying the experiences of people living with LC is essential to inform and improve care practice, as well as understanding that LC is a real health condition that is present in the lives of many people who have had COVID‐19 [20]. Based on the findings, it is necessary to increase communication and knowledge about LC in the healthcare and political spheres. All healthcare professionals must get training, due to the critical involvement of non‐physicians in triage and evaluation (e.g., nurses, community health agents), as well as training in the rehabilitation and management of LC [79]. Furthermore, as knowledge about persistent LC increases, we hope that there will be increased awareness among family members and employers to understand that persistent post‐COVID symptoms may still occur months or years after COVID‐19 infection, and impact personal and work life activities [80, 81]. Professionals’ perception of restricted care choices, coupled with inadequate surveillance or training from health system managers, contributes to a vicious cycle of under‐recognition of LC—cycle of invisibilization—in an under‐resourced and overburdened healthcare system in Brazil, hindering patients’ access to care [79].

It is essential to conduct additional longitudinal qualitative research that is comprehensive and considers the intricacies of living with a post‐viral disease like LC. In an ideal world, this type of research would support patients in developing a shared comprehension of the significance of life with this (and other) post‐viral illnesses. As the researcher restricted the use of theory to influence participants, patients were more dependent on themselves in this study. In light of our discoveries regarding the co‐construction of the narrative with the comprehension of others, additional research could involve interviewing the loved ones and caregivers of individuals with LC. Lastly, the legal acknowledgement of LC as a disability necessitates explicit diagnostic criteria substantiated by clinical research into the fundamental processes of enduring symptoms [82].

## 5 Conclusion

As far as we know, this was the first study to qualitatively understand the impact of persistent respiratory post‐COVID symptoms 12 months following SARS‐CoV‐2 infection. Participant experiences converged upon three categories: (1) imbalance between life before and after being infected by COVID‐19; (2) living with acute post‐COVID respiratory symptoms; and (3) struggling with persistent post‐COVID respiratory symptoms. These findings emphasize the intricate issues encountered by individuals with LC and reinforce the pressing necessity for focused interventions, research initiatives and social awareness efforts to meet the varied requirements and improve the quality of life for those with LC. Besides, these categories offer specific and practical recommendations for enhancing the experiences of individuals with LC respiratory symptoms while seeking services for symptom alleviation. Implementing these recommendations has significant potential to enhance care experiences for LC and may improve overall patient wellbeing. Moreover, LC patients require enhanced support due to problems such as scarce resources, uncertainty over the pandemic and its repercussions, and the substantial number of LC cases as COVID‐19 persists. There is an urgent need for additional resources for both research and practice to effectively address the requirements of individuals living with LC.

## Patient or Public Contribution

People living with Long COVID actively contributed to the study by participating in individual interviews and addressing pertinent topics to accurately capture their needs and experiences, thus contributing toward the development as of a person‐centred care approach.

## Author Contributions

**Giovana Alves Santos:** conceptualization, writing – original draft, methodology, writing – review and editing, data curation, formal analysis, visualization, and investigation. **Carlos Laranjeira:** project administration, conceptualisation, methodology, formal analysis, writing – original draft, writing – review and editing, visualization, investigation, supervision, and funding acquisition. **Lígia Carreira:** writing – review and editing. **Vanessa Denardi Antoniassi Baldissera:** writing – review and editing. **Maria Fernanda do Prado Tostes:** writing – original draft. **Viviani Camboin Meireles:** writing – original draft. **Rosella Santoro Ageno:** writing – review and editing. **Maria Aparecida Salci:** conceptualisation, writing – review and editing, supervision.

## Conflicts of Interest

The authors declare no conflicts of interest.

## Data Availability

The raw data supporting the conclusions of this article will be made available by the authors upon request.
